# *Scutellaria baicalensis *decreases ritonavir-induced nausea

**DOI:** 10.1186/1742-6405-2-12

**Published:** 2005-12-20

**Authors:** Han Aung, Sangeeta Mehendale, Wei-Tien Chang, Chong-Zhi Wang, Jing-Tian Xie, Chun-Su Yuan

**Affiliations:** 1 Tang Center for Herbal Medicine Research, Pritzker School of Medicine. The University of Chicago, IL 60637, USA; 2 Departments of Anesthesia & Critical Care. Pritzker School of Medicine. The University of Chicago, IL 60637, USA; 3 Committee on Clinical Pharmacology and Pharmacogenomics. Pritzker School of Medicine. The University of Chicago, IL 60637, USA

## Abstract

**Background:**

Protease inhibitors, particularly ritonavir, causes significant gastrointestinal disturbances such as nausea, even at low doses. This ritonavir-induced nausea could be related to its oxidative stress in the gut. Alleviation of drug-induced nausea is important in effectively increasing patients' compliance and improving their quality of life. Conventional anti-emetic drugs can only partially abate the symptoms in these patients, and their cost has also been a concern. Rats respond to nausea-producing emetic stimuli by increasing consumption of non-nutritive substances like kaolin or clay, a phenomenon known as pica. In this study, we used this rat pica model to evaluate the effects of *Scutellaria baicalensis*, a commonly used oriental herbal medicine, on ritonavir-induced nausea.

**Results:**

Rats treated with 20 mg/kg ritonavir significant caused increases of kaolin consumption at 24 to 48 hr (*P *< 0.01). Pretreatment with 0.3 and 3 mg/kg *Scutellaria baicalensis *extract significantly decreased ritonavir-induced kaolin intake in a dose-related manner (*P *< 0.01). Compared to vehicle treatment, the extract completely prevented ritonavir-induced kaolin consumption at dose 3 mg/kg. The area under the curves (AUC) for kaolin intake from time 0 to 120 hr for vehicle only, ritonavir only, SbE 0.3 mg/kg plus ritonavir, and SbE 3 mg/kg plus ritonavir were 27.3 g•hr, 146.7 g•hr, 123.2 g•hr, and 32.7 g•hr, respectively. The reduction in area under the curves of kaolin intake from time 0 to 120 hr between ritonavir only and SbE 0.3 mg/kg plus ritonavir, ritonavir only and SbE 3 mg/kg plus ritonavir were 16.0% and 77.7%, respectively.

**Conclusion:**

*Scutellaria baicalensis *significantly attenuated ritonavir-induced pica, and demonstrated a potential in treating ritonavir-induced nausea.

## Introduction

HIV infection/AIDS is a deadly disease that has affected over 30 million people world-wide and at least a million people in this country [1]. To achieve the treatment goal of reduction in viral loads and improved life expectancy, the treatment guidelines entail that patients be highly compliant to the drug regimens for an extended period of time [2,3]. However, the main obstacle that dissuades the patient from the goal is medication-induced side effects [2]. These side effects not only cause deterioration of the quality of life, but also have a major negative impact on compliance [4]. Nausea/vomiting is one such significant medication-related side effect that has been widely reported [5-10].

Protease inhibitor is a commonly used class of anti-HIV drugs [11,12]. Protease inhibitors, especially ritonavir, however, cause significant gastrointestinal disturbances, such as nausea/vomiting [7,8,11]. Although ritonavir can be used as a protease inhibitor by itself, it produces numerous side effects at the doses needed for an anti-viral effect, and thus it is poorly tolerated. Often ritonavir is used at a low dose to "boost" the drug levels of other protease inhibitors. Even at low doses, patients still could have ritonavir induced gastrointestinal side effects, especially nausea [12-14]. The incidence of ritonavir induced nausea/vomiting is approximately 23%, significantly higher than other antiretroviral agents [15]. Thus, alleviation of ritonavir-induced nausea could improve patients' compliance and their quality of life. Conventional anti-emetic drugs can only partially abate the symptoms in these patients, and their cost has also been a concern [16].

Rats react to nausea/vomiting stimuli by altered feeding habits, manifested as increased consumption of non-nutritive substances like kaolin (a type of clay), known as pica [17-20]. We previously demonstrated a significant anti-nausea (anti-pica) effect of anti-oxidant herbal medicines, i.e., *Scutellaria baicalensis *and ginseng, in cisplatin-induced pica in rats [21,22]. It has been shown that ritonavir caused endothelial cell cytotoxicity and oxidative stress, and this oxidative stress could be a mechanism of ritonavir-induced tissue dysfunction [23,24]. In this study, we used a rat model to investigate the role of anti-oxidant herb, *S. baicalensis *extract (SbE) in decreasing ritonavir-induced nausea.

## Methods

### Animals

The experimental protocol was approved by the Institutional Animal Care and Use Committee (IACUC) of the University of Chicago. Male Wistar strain rats (Harlan Sprague Dawley, Indianapolis, IN), weighing between 150–300 g, were used in this study. Animals were housed in standard isolation cages (45 cm × 35 cm × 25 cm) in environmentally controlled conditions with 12 hr light /12 hr dark cycle. Rats were allowed free access to water, standard laboratory rat chow (Harlan-Teklad, Madison, WI) and kaolin (see below), placed in separated containers continuously available throughout the experiment.

### Preparation and analysis of *S. baicalensis* extract (SbE)

*S. baicalensis *was obtained from a single batch from the Beijing Chinese Herbal Medicine Company. Voucher specimen was deposited in our laboratory at the Tang Center for Herbal Medicine Research, University of Chicago. The herbal sample was tested by Applied Consumer Services (Hialeah Gardens, FL), and it has been confirmed that the sample is free from contaminants such as microorganisms, pesticide residues, and toxic elements.

The root of *S. baicalensis *was soaked in cold water for 2 hr, and then cut into small pieces (less than 2 mm). These pieces were treated with hot water (approximately 95°C) for 1 hr. The filtrate obtained following the hot water treatment was evaporated under vacuum and lyophilized. During the experiment, the dried powder was dissolved in balanced salt solution (BSS), and centrifuged for 5 min (1,200 rpm). The supernatant was used.

**Figure 1:**
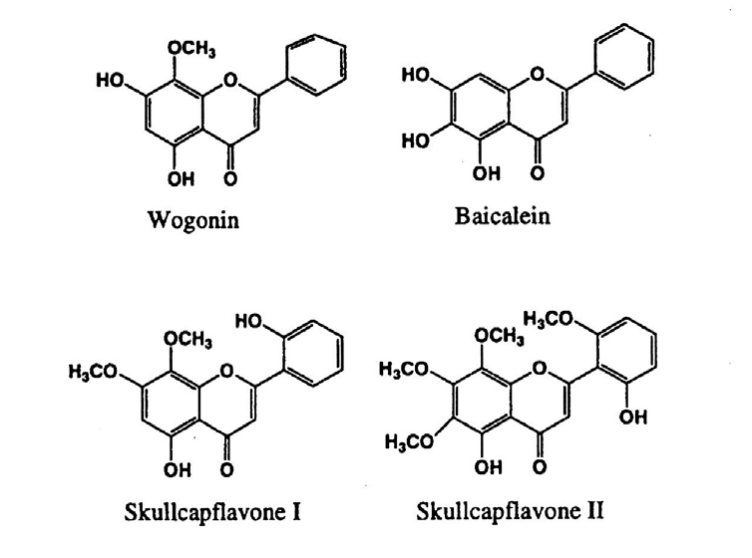
Structures of four major constituents isolated from *Scutellaria baicalensis *roots.

The extract was analyzed by liquid chromatography/mass spectrometry (LC/MS, Hitachi M1000, Hitachi Denshi, Ltd., Japan) with Atmospheric Pressure Chemical Ionization interface. The extract was dissolved in deionized water at a concentration of 1 mg/ml. The mobile phase was 14 mM ammonium acetate in acetonitrite (v/v:1/99) and flow rate was 0.8 ml/min. The sample (150 μl solution) was injected. The system was calibrated with flavopiridol. The extract contained the following flavones: wogon (51.5%), baicalein (35.6%), skullcapflavone I (4.8%), and skullcapflavone II (8.3%) (Figure 1) [25].

### Kaolin preparation

Kaolin was prepared based on the method described previously [21]. Briefly, pharmacological grade kaolin (or hydrated aluminum silicate; Fisher, Fair Lawn, NJ) and acacia (or Gum Arabic; Fisher, Fair Lawn, NJ) were mixed using a 99:1 ratio. Distilled water was used to form a thick paste of this mixture. The paste was rolled and cut into pieces that resembled regular rat chow pellets. The pellets were dried at room temperature for 72 hr.

### Experimental protocol

Rats placed in individual cages were allowed access to both regular food and kaolin during the 3-day adaptation period prior to study period. Rats then received ritonavir in the morning on 2 consecutive days (0 and 24 hrs) by oral gavage [26-28]. SbE and baicalein pretreatments were administered intraperitoneally [21], 30 min prior to each ritonavir administration. Rats were observed immediately and at 2 hr to ensure that animals are not distressed and they are comfortable.

During the experiment, kaolin and food pellets was weighed to the nearest 0.1 g and replaced in the containers every morning at the same time after collecting the remaining kaolin and food from the previous day. Kaolin and food intake was measured every 24 hr for 5 days.

The animals did not demonstrate any signs of adverse effects such as restlessness or respiratory distress following test administrations.

## Statistical analysis

Data were analyzed using a two-way analysis of variance (ANOVA) with group and time as the two factors. Statistical significance was considered at *P *< 0.05.

## Results

**Figure 2:**
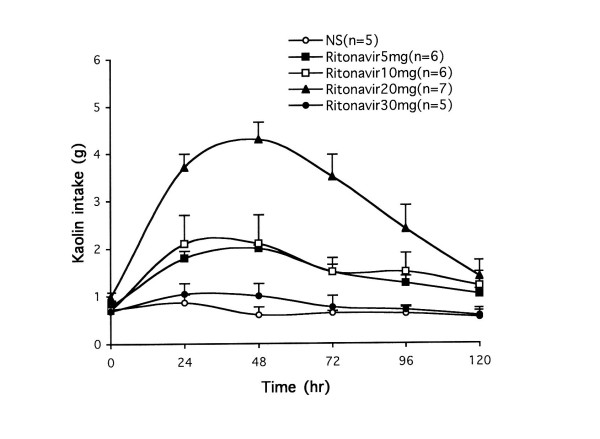
Effect of ritonavir (mg/kg) on kaolin intake in rats. Ritonavir doses at 5, 10 and 20 mg/kg induced significant increases of kaolin intake at 24 and 48 hr (*P *< 0.01), while 30 mg/kg did not further increase kaolin intake. NS = normal saline.

**Figure 3:**
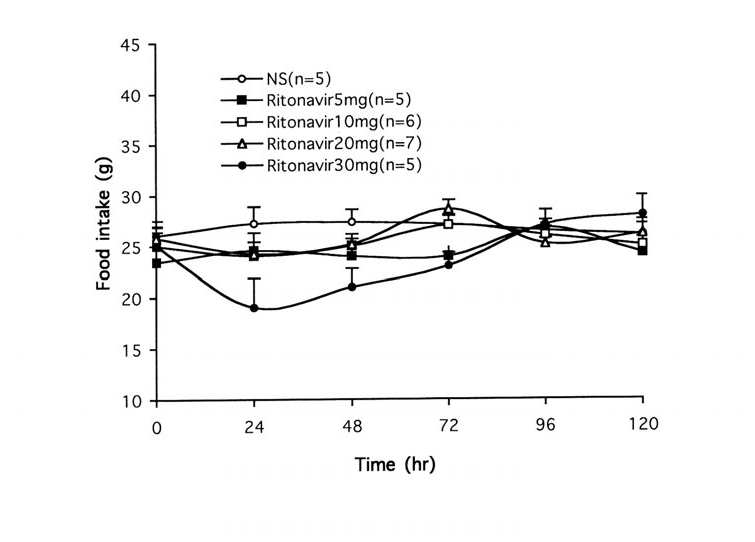
Effects of ritonavir (mg/kg) on food intake in rats. There was a significant reduction in food intake following 30 mg/kg ritonavir treatment at 24 and 48 hr (*P *< 0.05). NS = normal saline.

Rats treated with vehicle (normal saline) only consumed less than 1 g/day of kaolin during the consecutive 5 days (0, 24, 48, 72, 96, 120 hr), indicating that saline does not obviously induce pica (Figure 2). Figure 2 also showed effects of ritonavir on kaolin intake. Ritonavir doses at 5, 10 and 20 mg/kg significant caused increases of kaolin consumption at 24 to 48 hr in a dose-related manner (*P *< 0.01). Increasing ritonavir dose to 30 mg/kg did not further increase kaolin intake, suggesting toxic effect at higher ritonavir dose. This possible toxic effect was supported by the fact that at 30 mg/kg, ritonavir significantly reduced food intake at 24 to 48 hr (*P *< 0.05; Figure 3). Based on these results, we selected 20 mg/kg ritonavir dose for evaluation of herbal effects on the drug-induced pica.

**Figure 4:**
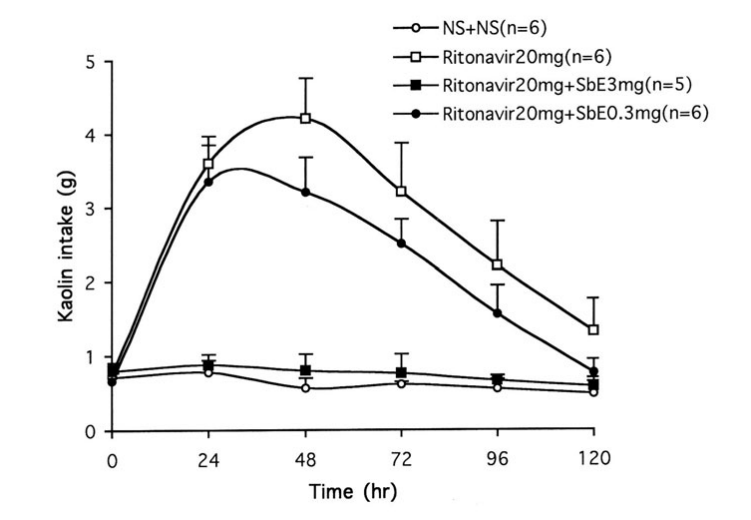
Effects of pretreatment with *Scutellaria baicalensis *extract (SbE, mg/kg) on kaolin intake induced by ritonavir in rats. Increased kaolin intake induced by ritonavir (mg/kg) was decreased with SbE pretreatment in a dose-related manner (*P *< 0.01). NS = normal saline.

Figure 4 demonstrated that pretreatment with SbE significantly decreased ritonavir-induced kaolin intake in a dose-related manner (*P *< 0.01). Compared to vehicle treatment, SbE at dose 3 mg/kg completely prevented ritonavir-induced kaolin consumption. The area under the curves (AUC) for kaolin intake from time 0 to 120 hr for vehicle only, ritonavir only, SbE 0.3 mg/kg plus ritonavir, and SbE 3 mg/kg plus ritonavir were 27.3 g•hr, 146.7 g•hr, 123.2 g•hr, and 32.7 g•hr, respectively. The reduction in AUC of kaolin intake between ritonavir only and SbE 0.3 mg/kg plus ritonavir, ritonavir only and SbE 3 mg/kg plus ritonavir were 16.0% and 77.7%, respectively. Food intake was not affected significantly in all these groups.

In addition, when SbE 3 mg/kg was administered alone, the extract did not affect kaolin consumption and food intake.

## Discussion

Typically, AIDS patients are treated with combinations of different drug groups to achieve the therapeutic goals of reduced viral loads and improved life expectancy [1]. Potent as these drugs may be, they are certainly not free from side effects [1], and thus add a challenging dimension to the management of drug-related adverse reactions. Patient adherence to the therapeutic regimen is of vital importance, as non-adherence can result in reduced suppression of viral load and also development of drug resistance [29]. Adherence of more than or equal to 95% is required to maintain control over viral replication, but in reality, the adherence rate is often lower with most studies showing that 40–60% of patients are less than 90% adherent [3]. Apart from other causes, drug-induced side effects are greatly responsible for poor patient compliance. Frequency and severity of side effects was one of the primary barriers to adherence, with nausea/vomiting topping the list of side effects [2]. Thus, any step towards improving patients' adherence to the drug regimen is vital for successful management of the disease.

The mechanism by which ritonavir causes nausea has not been investigated. Protease inhibitors cause premature cardiovascular diseases [23,30]. It has been shown that ritonavir induced cytotoxicity of human endothelial cells [23,30]. Conklin et al. [24] investigated the effects of ritonavir on vascular endothelial cell function in porcine carotid arteries. Superoxide and nitrotyrosine levels were measured. They observed that endothelium-dependent vasorelaxation was significantly reduced in ritonavir-treated vessels compared to controls. There were significant increases in basal and NADPH-stimulated superoxide production in vessel rings treated with ritonavir compared to control vessels. Dihydroethidium staining and nitrotyrosine staining were also elevated in endothelial cells of ritonavir-treated vessels, indicating increased superoxide production and increased oxidative stress, respectively, in ritonavir-treated vessels compared to controls. Their data demonstrated that increased oxidative stress could be a mechanism of ritonavir-induced cellular dysfunction.

In addition to ritonavir, nausea/vomiting is also associated with a number of other commonly prescribed drugs in AIDS pharmacotherapy [31,32]. A combination anti-retroviral therapy is advocated for initial treatment of established AIDS cases [1], and this multi-pharmacy increases the likelihood of emesis. Additionally, AIDS-related CNS disorders, gastroparesis, autonomic neuropathy and secondary infections can further aggravate drug-induced emesis [33]. Thus, anti-emetic use may have a wider scope in the management of AIDS patients, beyond ritonavir-induced nausea.

*S. baicalensis *is a widely used herb in traditional medical systems of China and Japan [34,35]. The dried root has been used clinically for allergies, inflammatory diseases, hyperlipidemia, and arteriosclerosis [34]. We previously observed that SbE attenuated cisplatin-induced emesis in rats. [34]. The major constituents in SbE are flavonoids, a group of polyhydroxy phenols [36]. These flavonoids, such as baicalein (Figure 1), possess anti-oxidant and other effects, which could also potentially be used to boost immune responses in HIV patients [37].

Several *in vivo *and *in vitro *studies have demonstrated the anti-oxidant capabilities of SbE. Kimuya et al. [36] and Kimura *et al*. [34] reported that liver homogenates of rats who were orally administered SbE, baicalein, and wogonin were resistant to lipid peroxidation. Gabrielska et al. [38] showed that an extract of SbE reduced liposomal lipid peroxidation. In a similar study, Gao et al. [39] observed that baicalein administration inhibited iron-dependent lipid peroxidation in rat liver microsomes. Subsequently, Gao et al. [40] showed that baicalein prevented cell death in fibroblasts damaged by H_2_O_2_, tetra-butyl hydroperoxide, and superoxide anions. Hamada et al. investigated *in vitro *radical scavenging effect of baicalein, and quantified superoxide and hydroxyl radical-scavenging effect by electron spin resonance spectrometry [41]. They reported that in a hypoxanthine-xanthine system, baicalein strongly reduced superoxide radicals. Our laboratory has reported that SbE dose-dependently attenuated effects of reactive oxygen species (ROS) in cardiomyocytes [25].

Ritonavir is known to induce nausea in significant number of AIDS patients [7,8,12,14] but has not been studied in an animal model. In this study, we observed that SbE attenuated kaolin intake or pica in ritonavir-treated rats. Our data support the hypothesis that ritonavir-induced emesis is related to its oxidative stress in the gut, and anti-oxidant herbs may have a therapeutic potential in ritonavir-induced emesis in AIDS patients.

An advantage of botanical therapies is the reduction of healthcare cost. With expensive anti-AIDS therapy, efficacious herbal products will bring down the cost of care, while providing the patient with relief from drug-induced side effects and improving their quality of life. AIDS patients are known to use herbal products more often than the general populace [1,42], and evidence-based herbal therapies can offer them with a practical alternative. Thus, systematic herbal evaluation, including conducting controlled clinical trials, is important in offering options to conventional pharmacological treatment [43].
